# Multiple Sclerosis in a Person With Hirschprung's Disease: A Case Report

**DOI:** 10.1002/ccr3.72984

**Published:** 2026-07-10

**Authors:** Masoud Etemadifar, Shima Shoeib, Mehri Salari, Nahad Sedaghat

**Affiliations:** ^1^ Alzahra Research Institute Alzahra University Hospital, Isfahan University of Medical Sciences Isfahan Iran; ^2^ School of Medicine Isfahan University of Medical Sciences Isfahan Iran; ^3^ Men's Health and Reproductive Health Research Center Shahid Beheshti University of Medical Sciences Tehran Iran

**Keywords:** EDNRB, genetic mutation, Hirschsprung's disease, multiple sclerosis

## Abstract

Multiple sclerosis (MS) is the most common demyelinating disease of the central nervous system. Hirschsprung's disease is a condition characterized by absence of nerves in the intestines, secondary to failure of migration of neural crest cells therein. Co‐occurrence of these conditions is reported previously in a family with a stop mutation in the EDNRB gene. In the present report we present the case of a 26‐year‐old man who presented with complaints of left facial weakness, episodic generalized headaches, and imbalance. He was previously diagnosed with Hirschsprung's disease and had undergone total colectomy. Subsequent evaluations confirmed MS. Family history was unremarkable, with no family members affected by either condition. Furthermore, we present our experience in managing the case with dimethyl fumarate disease‐modifying therapy.

## Key Clinical Message

A 26‐year‐old man with Hirschsprung's disease, previously treated surgically, presented with left facial weakness, imbalance, and a history of episodic headaches. Family history was unremarkable. MRI confirmed multiple sclerosis. Methylprednisolone pulse therapy resulted in near‐complete recovery. Subsequent disease‐modifying therapy with dimethyl fumarate was adequately‐efficacious and well‐tolerated, with no gastrointestinal complications.

## Graphical abstract

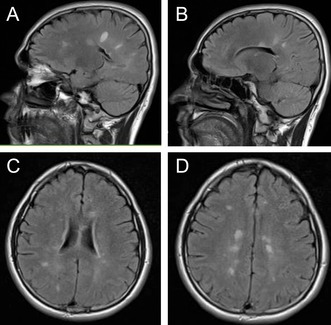

Sagittal and axial fluid‐attenuated inversion recovery (FLAIR) Brain MRI sequences reveal white matter hyperintensities consistent with multiple sclerosis in a person with Hirschprung’s disease.

## 1 Introduction

Multiple sclerosis (MS) is a disease usually affecting people in their 30s and characterized by demyelination of neurons in the central nervous system [1]. The people with MS (pwMS) could experience a wide range of conditions, such as sensorimotor deficits, fatigue, visual disturbances, cognitive impairment, and are at increased risk for psychiatric disorders [1]. As a result, MS imposes a great burden on affected people.

Hirschsprung's disease (HSCR) or congenital aganglionic megacolon, on the other hand, is a term that describes neurodevelopmental conditions characterized by lack of intestinal nerve ganglia (i.e., aganglionosis) particularly in the large intestine [2, 3, 4]. Based on the extension of the aganglionosis, patients with HSCR are often characterized as short‐segment (not extending beyond upper sigmoid, 75% of cases), long‐segment (extending beyond upper sigmoid, 20% of cases), total colonic aganglionosis (TCA, involving the entire colon, 3%–12% of cases), and total intestinal aganglionosis (involving the entire intestines, < 1% of cases) [3, 4]. Constipation and failure to pass meconium within 72 h of birth constitute the most common presenting symptoms of HSCR, mostly seen in the newborn and rarely in the prenatal period [3, 4, 5].

MS pathogenesis involves recurrent autoimmune demyelination and subsequent neuroaxonal damage in multiple, distinct areas of the CNS. MS etiology remains unknown, yet both genetic and environmental factors are associated with the risk of MS development [6] On the other hand, HSCR pathogenesis involves failure of neural crest cells in migrating to the intestines and forming the enteric nervous system. Mutations in the genes responsible for effective migration and placement of neural crest cells in the digestive tract are largely implicated in development of HSCR. For instance, loss of function mutations of the *RET* proto‐oncogene and the *EDNRB* gene are often associated with HSCR, [7] with the former seemingly causing more severe phenotypes such as TCA [8, 9].

Co‐occurrence of MS and HSCR is rarely reported. Recently, a whole exome sequencing study in a family with co‐occurring MS and HSCR [10] revealed a stop mutation in the *EDNRB*, suggesting a pathogenic role of this mutation causing both MS and HSCR. Sporadic co‐occurrence of MS and HSCR, on the other hand, which may involve an alternative process, is not reported to date, to the best of our knowledge. The co‐occurrence of MS and other diseases, especially genetic disorders, could reveal further avenues of research on the pathology of MS. Thus, in the present study we aimed to present the rare case of a person with severe HSCR—likely TCA—who was diagnosed with and treated for MS.

## 2 Case Presentation

### 2.1 Case History/Examination

A 26‐year‐old man presented to the Isfahan MS clinic, Iran, complaining of left facial weakness, generalized headaches, and imbalance. Similar episodes were also experienced previously, which resolved spontaneously after a few days. Their past medical history was furthermore, particularly notable for HSCR, diagnosed in infancy via full‐thickness rectal biopsy following recurrent episodes of severe bowel obstruction (with symptoms including vomiting, abdominal distension, and constipation). The patient underwent several surgeries until the age of 18, including several segmental colon resection operations. However, he continued to experience persistent obstructive symptoms, including abdominal distension, chronic constipation, and intermittent gastroenteritis with nausea and vomiting—suggestive of extensive aganglionosis and failure of segmental surgeries. Therefore, he ultimately underwent total colectomy with ileostomy placement at the age of 18 to manage ongoing and refractory gastrointestinal symptoms. According to the patient, pathology at that time was consistent with a severe form of HSCR—likely TCA.

Furthermore, familial history was unremarkable, with no history of MS and/or HSCR in their first, second, and third‐degree kin. Physical examination was likewise unremarkable. A comprehensive neurological examination, however, revealed horizontal nystagmus, left peripheral facial weakness, increased lower extremity deep tendon reflexes bilaterally, and right‐sided Babinski sign.

### 2.2 Differential Diagnosis

The clinical picture most strongly favored a CNS inflammatory demyelinating disorder, particularly MS because the person was a young adult with recurrent episodes of neurological dysfunction that resolved spontaneously, along with signs of brainstem and upper motor neuron spinal cord involvement suggesting dissemination in space and time. Myelin oligodendrocyte glycoprotein antibody‐associated disease, neuromyelitis optica spectrum disorder, and neurosarcoidosis were further considered as important differential diagnoses that can also present with relapsing neurological deficits. The unusual Hirschsprung history, in addition, warranted the consideration of an underlying genetic neurocristopathy.

### 2.3 Investigations and Treatment

Brain and cervical spine MRI were performed before and after gadolinium administration, revealing multiple fluid‐attenuated inversion recovery (FLAIR)‐hyperintense lesions in the periventricular, subcortical and juxtacortical regions of the brain, as well as corpus callosum, midbrain, pons, medulla oblongata, and cerebellum (Figure 1). Some lesions showed gadolinium enhancement as well, in T1‐weighted sequences, and cervical spinal cord was also involved (Figure 2). The dissemination in space and time was, therefore, established without lumbar puncture, and the diagnosis of relapsing–remitting MS (RRMS) was made according to the 2017 McDonald criteria.

**FIGURE 1:**
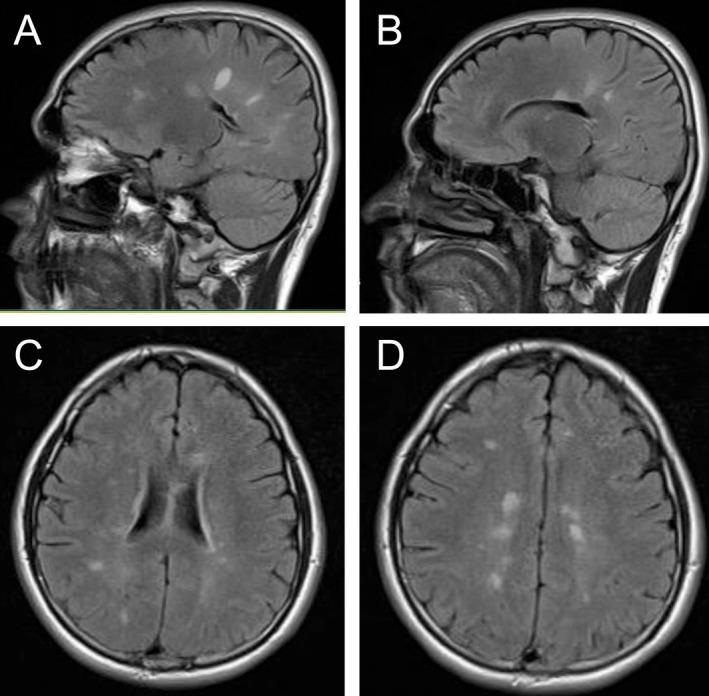
Brain MRI. Sagittal (A and B) and axial (C and D) fluid‐attenuated inversion recovery (FLAIR) sequences reveal multiple white matter non‐space‐occupying hyperintensities distributed across various regions, including the periventricular, subcortical and juxtacortical regions, brainstem and the cerebellum. The morphological appearance of these hyperintensities, for example, the “Dawson finger” appearance of the periventricular lesions, is consistent with multiple sclerosis.

**FIGURE 2:**
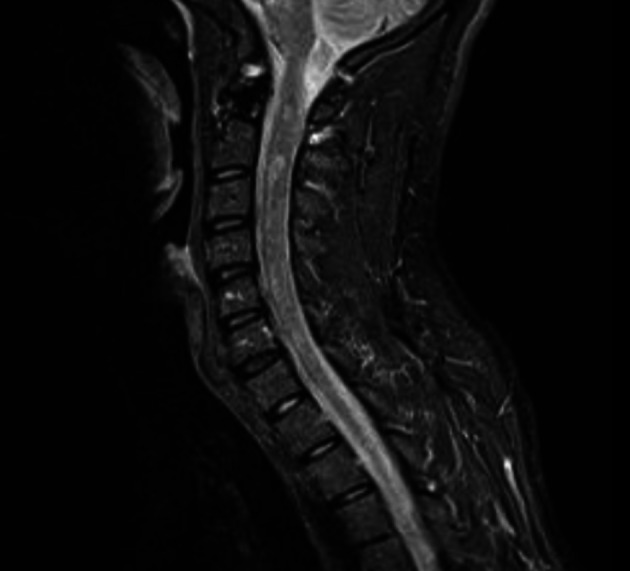
Cervical spinal cord MRI. Sagittal fluid‐attenuated inversion recovery (FLAIR) sequences show multiple hyperintensities in the cervical spinal cord.

Methyl prednisolone pulse therapy was given for three days, and then tapered to discontinuation. This resulted in near‐complete resolution of the neurological deficits. The patient was offered disease‐modifying therapy (DMT) with dimethyl fumarate, which is well tolerated up to the present. Of note, no gastrointestinal complications occurred, and his ileostomy continues to function properly.

### 2.4 Outcome and Follow‐Up

In their latest follow‐up visit (November 2025), the person had some residual neurological deficits, mild facial hemiparesis. Yet, he did not experience any subsequent clinical or radiological relapses and has had a stable disability status since his diagnosis and treatment. Their expanded disability status scale score was 1.5 in his last follow‐up visit.

Regardless of this severe condition, the patient tolerated treatment with dimethyl fumarate and none of the gastrointestinal side effects for example, nausea or abdominal pain occurred. The patient continues to receive multidisciplinary care, including neurology, gastroenterology, and rehabilitation services, to address the complex and varied aspects of his condition.

## 3 Discussion

We presented a case of coexisting MS in a person with severe HSCR. Of note, this is the first report of such coexistence in a sporadic case to the best of our knowledge. The well tolerability of dimethyl fumarate in a person with HSCR as well as its good clinical efficacy could guide management of such rare cases in the future.

From a pathophysiological perspective, MS is a multifaceted disease which can be characterized by immune‐mediated demyelination and neurodegeneration within the CNS [11]. The etiology of MS contains a combination of genetic susceptibility, environmental factors, and immune system dysregulation [12]. HSCR, in contrast, is a congenital state following the failure of neural crest cells to migrate throughout the intestinal development, leading to aganglionic (and as a result, severely hypomotile) segments mostly in the colon [13]. As previously mentioned, genetic mutations, above all in the *RET* proto‐oncogene and the *EDNRB* gene, are primary drivers of HSCR [3, 4, 14].

The concurrent presentation of MS and HSCR in the presented case could point towards an intricate relationship between the central and enteric nervous systems, suggesting a potential genetic overlap between these conditions. Granström et al. [10], notably, discovered a novel stop mutation in the *EDNRB* (c.C397T, p.R133X) in a family with both HSCR and MS. The *EDNRB* encodes the Endothelin B receptor, a vital G protein‐coupled receptor for the development of neural crest‐derived cells [15, 16]. Mutations in this gene are well‐documented in association with HSCR as well as some other neurocristopathies [15, 16]. The presence of the novel *EDNRB* mutation in that family in absence of other MS‐associated genotypes may suggest that the *EDNRB* might have broader implications beyond what is currently known, potentially affecting neural and immune functions more generally [17]. The dual occurrence of HSCR and MS in the presented sporadic case further raises the possibility of a shared pathogenic mechanism in MS and HSCR warranting further investigation [18]. The severity of HSCR in this case may furthermore, suggest (at least) a *RET* mutation, as *RET* mutations seems to occur in the majority of TCA cases; [8, 9] this remains to be investigated in the future.

This case report has several limitations. First, the absence of genetic analysis in our patient limits our ability to explore the potential genetic origin of the co‐occurrence of HSCR and MS. In future studies, exploring genetic attributes could provide more robust information on potentially shared biological pathways underlying these diseases. Second, the rarity of the co‐occurrence of these diseases limits the generalizability of our findings. While our case contributes to the existing literature, it is based on a single patient. Larger studies are needed to confirm any potential links between HSCR and MS.

## 4 Conclusion

In conclusion, while our case adds to the developing body of knowledge as regards the co‐occurrence of HSCR and MS, further research is warranted to discover the potentially mutual genetic and immunological underpinnings of these diseases. The presented case further highlights the importance of considering rare comorbidities in clinical practice, as well as the need for continued investigation into the complex interactions between genetic, environmental, and immunological factors in neurodevelopmental and neurodegenerative diseases. In addition, the present report constitutes initial evidence of the safety of MS treatments, particularly methylprednisolone pulse therapy and dimethyl fumarate DMT in the context of coexisting HSCR and/or total colectomy with ileostomy placement, informing and/or piloting further more robust studies on the subject.

## Author Contributions

**Masoud Etemadifar:** conceptualization, project administration, resources, supervision, writing – review and editing. **Shima Shoeib:** data curation, investigation, writing – original draft. **Mehri Salari:** resources, supervision, writing – review and editing. **Nahad Sedaghat:** validation, writing – original draft, writing – review and editing.

## Funding

The authors have nothing to report.

## Ethics Statement

Case reports, including the present report, do not require regional ethics committee approval according to the national research ethics guidelines. The subject of the present report provided their written informed consent for the reporting and publication of their anonymized medical case along with any unidentifiable images.

## Consent

The subject of the present report provided their written informed consent for the reporting and publication of their anonymized medical case along any unidentifiable images.

## Conflicts of Interest

The authors declare no conflicts of interest.

## Data Availability

All anonymized data produced during the present study is provided in the manuscript.
